# Neuroimaging alterations in dementia with Lewy bodies and neuroimaging differences between dementia with Lewy bodies and Alzheimer's disease: An activation likelihood estimation meta‐analysis

**DOI:** 10.1111/cns.13775

**Published:** 2021-12-06

**Authors:** Wen‐ying Ma, Min‐jie Tian, Qun Yao, Qian Li, Fan‐yu Tang, Chao‐yong Xiao, Jing‐Ping Shi, Jiu Chen

**Affiliations:** ^1^ Department of Neurology Affiliated Nanjing Brain Hospital, Nanjing Medical University Nanjing Jiangsu China; ^2^ Department of Radiology Affiliated Nanjing Brain Hospital, Nanjing Medical University Nanjing Jiangsu China; ^3^ Institute of Neuropsychiatry Affiliated Nanjing Brain Hospital, Nanjing Medical University Nanjing Jiangsu China; ^4^ Institute of Brain Functional Imaging Affiliated Nanjing Brain Hospital, Nanjing Medical University Nanjing Jiangsu China

**Keywords:** dementia with Lewy bodies, neuroimaging, anatomical/activation likelihood estimation, coordinate‐based meta‐analysis, functional meta‐analytic connectivity modeling

## Abstract

**Aims:**

The aim of this study was to identify brain regions with local, structural, and functional abnormalities in dementia with Lewy bodies (DLB) and uncover the differences between DLB and Alzheimer's disease (AD). The neural networks involved in the identified abnormal brain regions were further described.

**Methods:**

PubMed, Web of Science, OVID, Science Direct, and Cochrane Library databases were used to identify neuroimaging studies that included DLB versus healthy controls (HCs) or DLB versus AD. The coordinate‐based meta‐analysis and functional meta‐analytic connectivity modeling were performed using the activation likelihood estimation algorithm.

**Results:**

Eleven structural studies and fourteen functional studies were included in this quantitative meta‐analysis. DLB patients showed a dysfunction in the bilateral inferior parietal lobule and right lingual gyrus compared with HC patients. DLB patients showed a relative preservation of the medial temporal lobe and a tendency of lower metabolism in the right lingual gyrus compared with AD. The frontal‐parietal, salience, and visual networks were all abnormally co‐activated in DLB, but the default mode network remained normally co‐activated compared with AD.

**Conclusions:**

The convergence of local brain regions and co‐activation neural networks might be potential specific imaging markers in the diagnosis of DLB. This might provide a pathway for the neural regulation in DLB patients, and it might contribute to the development of specific interventions for DLB and AD.

## Graphical abstract

This study shows that the brain structure and function damage of dementia with Lewy body is selective and specific, which is significantly different from that of AD. The frontal‐parietal, salience, and visual networks were all abnormally co‐activated in DLB, but the default mode network remained normally co‐activated compared with AD.

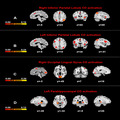

## 1 INTRODUCTION

Dementia with Lewy bodies (DLB) is characterized by fluctuating cognition, recurrent visual hallucinations, rapid eye movement sleep behavior disorder, and spontaneous parkinsonism,^1^ accounting for 15%–20% of the total dementia cases at autopsy.^2^, ^3^ Although DLB is the second most common neurodegenerative disorder after AD, the sensitivity of its diagnosis in clinical practice is suboptimal. The widely spread pathologies related to Lewy bodies and coexisting AD‐type pathologies^4^, ^5^, ^6^ make the clinical manifestations complex and highly variable, increasing the difficulty of the differential diagnosis between DLB and AD, especially in the early stages. Multimodal neuroimaging is widely used in clinical practice. For example, the role of DAT imaging in distinguishing DLB from AD is well established, with a sensitivity of 78% and specificity of 90%.^7^ A neuropathologically confirmed study showed that DAT imaging can distinguish between DLB and AD more accurately than the consensus clinical criteria.^8^ However, broader structural and functional studies provided conflicting results. Therefore, stable and consistent indicators that provide a theoretical basis for the diagnosis and differential diagnosis of DLB are still lacking.

Structural imaging can reflect changes in brain volume at voxelwise level.^9^, ^10^ Some reports showed the cortical atrophy of the frontal lobe,^11^ temporal lobe,^11^, ^12^ parietal lobe, and occipital lobe^12^ in DLB. However, other studies found a relatively concentrated pattern of atrophy in the subcortical brain, including midbrain, hypothalamus/thalamus, basal ganglia,^13^, ^14^ and substantia innominate.^15^, ^16^, ^17^ DLB patients with a similar level of dementia have relatively better preservation of the hippocampus, temporal lobe,^12^, ^14^, ^18^ and amygdala.^11^, ^19^ This aspect means that they are more likely to develop subcortical atrophy than AD patients.^13^, ^14^ A functional imaging report showed a hypoperfusion in the frontal, insular, and temporal cortexes of DLB patients, as well as the hypoperfusion in the parietal and temporoparietal cortexes of AD patients.^20^ Another article revealed that the temporal cerebral blood flow in DLB patients remained unchanged.^21^ Additionally, a reduced metabolic activity in the frontal and occipital lobes is observed in both DLB and AD, although more reduced in the former^22^, ^23^ Therefore, it is necessary to focus on these different findings to better understand the relatively uniform damage of brain regions.

Growing evidence suggests that neurodegenerative diseases are caused by brain network dysfunction rather than the dysregulation of an isolated brain region.^24^, ^25^ Local brain regions that are selectively damaged act as “nodes” in functional networks, representing the basis of the network degradation hypothesis.^26^ Brain network abnormalities detected in patients with DLB are predominantly described in the default mode network (DMN),^27^ frontal‐parietal network (FPN),^28^, ^29^ basal ganglia network,^30^, ^31^ and visual network (VIS).^32^ Therefore, functional meta‐analytic connectivity models (fMACM)^33^ should be further constructed based on locally convergent brain regions. This might allow to test the network degradation hypothesis in DLB and evaluate whether the regional degeneration in DLB reflects distinct human neural network architecture. Patterns of the involved neural networks might be used as predictors of disease‐related changes, thus providing a reference for the development of novel therapies, such as transcranial magnetic stimulation for network regulation.

Anatomical/activation likelihood estimation (ALE) is a powerful coordinate‐based meta‐analysis allowing to quantify consistent imaging findings across studies.^34^ The fMACM can be used to determine which brain regions are co‐activated above chance, with a particular seed region. The whole‐brain co‐activation pattern can be regarded as a surrogate for functional connectivity (FC).^35^, ^36^ A previous meta‐analysis investigated gray matter atrophy in DLB, but this investigation was limited to structural imaging.^37^ Currently, there is no consensus on brain structure and function damage in DLB patients, and whether the functional neural networks are dependent on the affected brain regions.

In this work, a quantitative meta‐analysis was performed to delineate the most affected brain regions in DLB patients to highlight the differences in imaging findings between DLB and AD. The fMACM technique was then used to identify the neural networks involved in the affected brain regions in DLB. According to previous studies, our hypothesis was that DLB is characterized by a convergent damaged brain regions compared with HCs and AD. Our specific expectation is to observe that the co‐activated neural networks prominently include the DMN, FPN, and VIS. Finally, the applications of some of the promising novel imaging modalities in DLB were reviewed, which may provide further insights into DLB pathophysiology.

## 2 METHODS

### 2.1 Literature search and study selection

The meta‐analysis was preregistered on Prospero (registration number: CRD42020162018) and was conducted according to the PRISMA statement.^38^ A systematic search was conducted on March 27, 2021, using PubMed, Web of Science, OVID, Science Direct, and Cochrane Library database using the following keywords: "Magnetic Resonance Imaging" [Mesh] OR "Positron‐Emission Tomography" [Mesh] OR "Tomography, Emission‐Computed, Single‐Photon" [Mesh] OR MRI OR “magnetic resonance imaging” OR “imaging” OR “neuroimaging” OR “brain imaging” OR “gray matter” OR “white matter” OR “voxel‐based morphometry” OR “VBM” OR “voxelwise” OR “positron emission tomography” OR PET OR “single photon emission computed tomography” OR SPECT AND Lewy OR "Lewy Body Disease" [Mesh] (Table S1). The reference lists of the eligible articles and relevant review articles were also screened to find potential additional studies. Authors not providing the necessary data were contacted to obtain clarification regarding the missing or unclear information.

The original studies included in this work were based on the following criteria: (1) they were published in English with peer review; (2) they report structural and functional neuroimaging changes related to the comparison between DLB patients and HCs (DLB‐HCs), or comparison between DLB and AD (DLB‐AD); (3) they report the whole‐brain results in three‐dimensional coordinates (x, y, z) in standard reference space (Talairach/Montreal Neurological Institute, MNI); and (4) they report the statistical significance. Structural imaging refers to the whole‐brain analysis using Voxel‐based morphometry (VBM). The functional imaging included the fludeoxyglucose positron emission tomography (FDG‐PET) and single‐photon emission computed tomography (SPECT). If the data from one study overlapped with those of another study, the largest group was selected for our meta‐analysis.

Studies with one of the following parameters were excluded: (1) the necessary data could not be obtained; (2) studies based on the analysis of the correlation between imaging indicators and clinical or biological indicators; (3) studies based on the analysis of the region of interest (ROI); and (4) studies that performed small volume correction.

**FIGURE 1:**
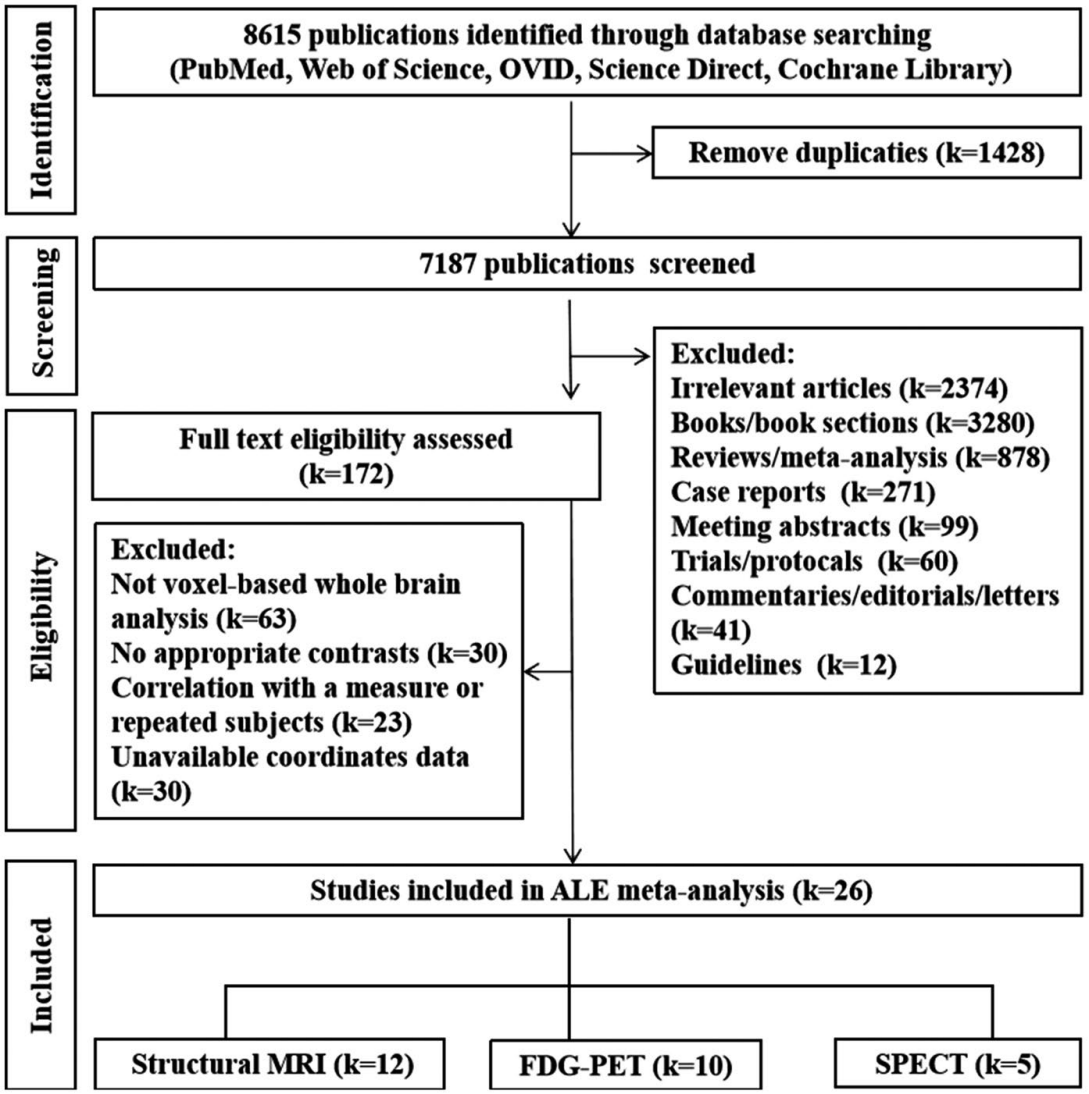
Flowchart of literature search and selection strategy. ALE, Anatomical/activation likelihood estimation; MRI, magnetic resonance imaging; FDG‐PET , fludeoxyglucose positron emission tomography; SPECT , single‐photon emission computed tomography. One study employed VBM and PET at the same time

Study selection, data extraction, and cross‐check were conducted by two researchers independently. Inconsistencies were resolved by discussion or by the involvement of a third reviewer. The flowchart of the literature search and selection strategy is shown in Figure 1.

### 2.2 Data extraction

The three‐dimensional coordinates, literature basic information, demographic data, and the experimental and imaging details were extracted from the eligible articles. Then, any coordinate (focus) reported in Talairach space was converted to MNI standard space using the Ginger ALE convert tool icbm2tal transformation.^39^ Each three‐dimensional coordinate is considered as a focus. Two authors (Wen‐ying Ma and Qun Yao) performed the data extraction independently.

### 2.3 Quality assessment

The quality of the included studies was assessed using a 12‐point checklist (Table S2). The checklist focused on three aspects in each study: (1) clinical and demographic characteristics of the samples; (2) imaging‐specific methodology; and (3) standardization of the results and conclusions. This checklist was based on previous meta‐analysis studies,^40^, ^41^ but it was modified to reflect key variables that are important to evaluate VBM or PET/SPET studies. Although the checklist was not designed as an evaluation tool, it provided some objective indication of the rigor of the individual studies. At least two authors reviewed each article and independently determined the integrity rating. A consistent score was obtained after discussion for article with inconsistent scores. The quality score of each study is shown in Table 1.

**TABLE 1:** Summary of studies included in ALE meta‐analysis

N	study	Manufacturer	Sequence	Field, T (coil, channels)	Thickness (mm)	Voxel Size (mm)	Matrix Size	FOV (mm)	FWHM (mm)	Modality	Contrasts (No. of foci)	Threshold *p* < (cor/uncor)	Standard Template	Quality scores (out of 12)
Structural image														
1	Burton et al., 2002^11^	Siemens	3D MPRAGE	1 (NA)	1	2 × 2 × 2	256 × 256	256	10	MRI (VBM)	DLB <HC (10); AD <DLB (5)	*p* < 0.05 (cor)	Talairach	11
2	Brenneis et al., 2004^127^	Siemens	3D FLASH	1.5 (NA)	1.5	1 × 1 × 1	256 × 256	230	8	MRI (VBM)	DLB <HC (7)	*p* < 0.05 (cor)	Talairach	10
3	Ishii et al., 2007^55^	General Electric	3D SPGR	1.5 (NA)	1.5	NA	NA	NA	12	MRI (VBM)	DLB <HC (2); DLB <AD (1)	DLB <HC: *p* < 0.05 (cor); DLB <AD: *p* < 0.001 (uncor)	Talairach	11.5
4	Sanchez‐Castaneda et al., 2009^128^	Philips	NA	1.5 (NA)	NA	0.98 × 0.98 × 1.3	NA	NA	8	MRI (VBM)	DLB <HC (4);	*p* < 0.05 (FWE)	Talairach	12
5	Takahashi et al., 2010^129^	General Electric	3D SPGR	1.5 (NA)	1.5	NA	256 × 256	220	6	MRI (VBM)	DLB <HC (3); AD <DLB (8)	*p* < 0.001 (cor)	Talairach	11
6	Watson et al., 2012^12^	Philips	3D MPRAGE	3 (8)	1	NA	240 × 216 × 180	NA	8	MRI (VBM)	DLB <HC (9); DLB <AD (3)	*p* < 0.05 (FWE)	MNI	12
7	Borroni et al., 2015^130^	Siemens	3D MPRAGE	1.5 (NA)	1	1 × 1 × 1	NA	250 × 250	10	MRI (VBM)	DLB <HC (9)	*p* < 0.001 (FDR)	Talairach	11.5
8	Blanc et al., 2016^131^	Phillips /Siemens	3D MPRAGE	3 (8/32)	1	1 × 1 × 1	240 × 240 × 180 /192 × 192 × 176	NA	8	MRI (VBM)	Pro‐DLB <HC (13); Pro‐AD <Pro‐DLB (1)	*p* < 0.05 (FWE)	MNI	12
9	Heitz et al., 2016^132^	Siemens	T1WSE	3 (NA)	1	1 × 1 × 1	NA	192	8	MRI (VBM)	DLB <HC (12); AD <DLB (14); DLB <AD (2)	*p* < 0.001 (uncor)	MNI	12
10	Peraza et al., 2016^133^	Philips	3D MPRAGE	3 (NA)	1	1 × 1 × 1	NA	240 × 240	8	MRI (VBM)	DLB <HC (1)	*p* < 0.05 (FWE)	MNI	11.5
11	Roquet et al., 2017^134^	Siemens	3D MPRAGE	3 (32)	NA	1 × 1 × 1	192 × 192 × 176	NA	8	MRI (VBM)	Pro‐DLB <HC (4); mild DLB <HC (1); mild AD <mild DLB (1)	*p* < 0.05 (cor)	MNI	12
12	Nemoto et al., 2021^135^	NA	NA	NA	NA	NA	NA	NA	8	MRI (VBM)	DLB <HC (6)	*p* < 0.05 (FWE)	MNI	12
Functional imaging														
PET														
1	Imamura et al., 1997^109^	NA	NA	NA	11	NA	NA	NA	10	PET ([18F]FDG) 185–259 MBq	DLB <AD (5); AD <DLB (4)	*p* < 0.01 (unclear)	Talairach	10.5
2	Ishii et al., 2007^55^	NA	NA	NA	NA	NA	128 × 128	NA	12	PET ([18F]FDG) 185–370 MBq	DLB <HC (10); DLB <AD (7)	DLB <HC: *p* < 0.05 (cor); DLB <AD: *p* < 0.001 (uncor)	Talairach	11
3	Perneczky et al., 2007^136^	Siemens	NA	NA	NA	2	128 × 128	NA	12	PET ([18F]FDG) 370 MBq	DLB <HC (6)	*p* < 0.05 (FDR)	Talairach	11
4	Yong et al., 2007^137^	General Electric	NA	NA	3.27	3.9	128 × 128	NA	16	PET ([18F]FDG) 300 MBq	DLB <HC (10);	*p* < 0.001 (uncor)	MNI	11
5	Teune et al., 2010^138^	Siemens	NA	NA	NA	1 × 1 × 1	NA	NA	10	PET ([18F]FDG) 200 MBq	DLB <HC (14); HC <DLB (18)	*p* < 0.05 (cor)	MNI	10.5
6	Iizuka et al., 2016^111^	Siemens	NA	NA	NA	NA	NA	NA	5	PET ([18F]FDG) 185 MBq	DLB <AD (3); DLB >AD (5)	*p* < 0.001 (uncor)	MNI	10
7	Iizuka et al., 2017^139^	Siemens	NA	NA	NA	NA	NA	NA	5	PET ([18F]FDG) 185 MBq	DLB <HC (3)	*p* < 0.001 (uncor)	MNI	10.5
8	Liu et al., 2017^140^	General Electric	NA	NA	4.25	2.5 × 2.5	128 × 128	NA	10	PET ([18F]FDG) 250 MBq	DLB <HC (29)	*p* < 0.001 (uncor)	MNI	10
9	Liguori et al., 2019^141^	General Electric	NA	NA	NA	NA	256 × 256	NA	NA	PET ([18F]FDG) 185–250 MBq	DLB <HC (6)	*p* < 0.05 (FDR)	Talairach	11
10	Iizuka et al., 2020^142^	Siemens	NA	NA	NA	NA	NA	NA	5	PET ([18F]FDG) 185 MBq	DLB <HC (3); DLB >HC (3); DLB <AD (2); DLB >AD (4)	NA	MNI	10.5
SPECT														
1	Colloby et al., 2002^94^	CamStar	NA	NA	5.4	5.4	64 × 64	NA	16	SPECT (99mTc‐HMPAO) 500 MBq	DLB <HC (8); DLB <AD (4)	DLB <HC: *p* < 0.05 (cor); DLB <AD: *p* < 0.01 (unor)	Talairach	12
2	Firbank et al., 2003^58^	NA	NA	NA	3.54	NA	128 × 128	NA	10	SPECT (99mTc‐HMPAO) 500 MBq	DLB <HC (2)	*p* < 0.001 (uncor)	Talairach	11
3	Takahashi et al., 2010^143^	NA	NA	NA	8	NA	64 × 64	NA	16	SPECT	DLB‐P < HC (3); DLB‐nP <HC (3)	*p* < 0.05 (cor)	Talairach	11
4	Misch et al., 2014^144^	Phillips	NA	NA	3.56	2 × 2 × 2	128 × 128	NA	12	SPECT (99mTc‐ECD) 740 MBq	DLB <HC (8)	*p* < 0.05 (FWE)	Talairach	12
5	Park et al., 2018^82^	Siemens	NA	NA	NA	2.1 × 2.1 × 3.9	128 × 128 × 47	NA	16	SPECT (99mTc‐HMPAO) 925 MBq	DLB <HC (14)	*p* < 0.01 (uncor)	Talairach	11.5

Note

All functional imaging experiments were in resting state.

Abbreviations: 99mTc‐ECD, technetium‐99 methyl cysteinate dimer; 99mTc‐HMPAO, technetium 99m‐hexamethylpropyleneamine oxime; AD, Alzheimer's disease; ALE, Anatomical/activation likelihood estimation; ASL, arterial spin labeling; cor, corrected; CTh, cortical thickness; DLB, dementia with Lewy bodies; DLB‐nP, DLB patients without parkinsonism; DLB‐P, dementia with Lewy bodies with parkinsonism; EPI, T2‐weighted echo planar; FDG‐PET, [18 F]fludeoxyglucose positron emission tomography; FLASH, fast low‐angle shot; fMRI, functional magnetic resonance imaging; FOV, field of view; FSPGR, fast spoiled gradient recalled echo; GE‐EPI, gradient echo echo‐planar imaging; HC, healthy controls; MPRAGE, magnetization‐prepared rapid acquisition gradient echo; MRI, magnetic resonance imaging; N, study number; NA, data not available; PET, positron emission tomography; pro‐AD, prodromal Alzheimer's disease; pro‐DLB, prodromal dementia with Lewy bodies; SPGR, spoiled gradient echo imaging; T1‐TFE, T1 turbo field echo; T1WSE, T1‐weighted spin echo sequences; uncor, uncorrected; VBM, voxel‐based morphometry.

### 2.4 Anatomical/activation likelihood estimation meta‐analysis

The ALE meta‐analysis was carried out in MNI space using the Ginger ALE software V3.0.2 (http://www.brainmap.org).^42^, ^43^ First, the MNI coordinates and sample size of each study were imported into Ginger ALE through a text file. The ALE algorithm treats each focus as Gaussian probability distributions centered at the given coordinates, to account for errors in spatial localization. The full width at half maximum (FWHM) of the Gaussian distributions was set according to the random‐effects approach, allocating tighter and taller Gaussian functions for larger sample sizes.^42^ Therefore, the ALE results were more reasonably weighted to larger sample size studies. Subsequently, the probabilities of all set foci were combined for each voxel, resulting in a modeled anatomical (MA) effects map. Then, one voxelwise ALE‐map was yielded by taking the union across these MA‐maps. ALE values represented the likelihood of convergent findings in different brain regions. The significance of the convergence results was determined by a permutation test comparing the ALE‐maps with an empirical null distribution. The resulting significant convergence results were labeled according to the probability cytoarchitecture map of the human brain, in which each voxel belonged to the most likely cytoarchitecture region.^44^ The maps were corrected with a cluster forming a threshold of *p *< 0.001 and cluster‐level family‐wise error (FWE) threshold at *p *< 0.05. Significance was tested using 1000 permutations. In addition, an extent threshold of 300 mm^3^ was applied. The final ALE‐maps were visualized with the MRIcron software (http://www.mricro.com).

Four separate ALE analyses were conducted: (1) structural imaging between DLB and HCs (n = 287; 77 foci; 13 experiments); (2) functional imaging between DLB and HCs (n = 256; 119 foci; 14 experiments); (3) structural imaging between DLB and AD (n = 160; 32 foci; 6 experiments); and (4) functional imaging between DLB and AD (n = 136; 23 foci; 5 experiments).

### 2.5 Jackknife sensitivity analysis

After the ALE analysis, a jackknife sensitivity analysis was performed by iteratively repeating the same analyses, but one dataset each time was excluded to test the replicability of the results across studies.^45^, ^46^, ^47^ A substantial variability suggests that the results are driven by specific studies that were ignored, thus compromising the robustness against spurious findings.

### 2.6 Fail‐safe N analysis

Traditional detection methods including size meta‐analysis are not suitable for the ALE method in order to consider the possibility of publication bias.^48^ Therefore, the potential publication bias in this study was evaluated by a post hoc noise simulation, which was referred to a modified version of the fails‐safe N (FSN) method.^49^ It was applied for the estimation of the robustness against unpublished neuroimaging findings. A recent study using the data from BrainMap provides evidence for the existence of a file drawer effect, with the rate of missing contrasts estimated as at least 6 per 100 reported.^50^ Therefore, the convergence meta‐analysis was retested starting with an additional 6% noise to evaluate the robustness of the identified clusters. The surviving clusters were then retested, with a noise rate of up to 30%, as in the previous study.^51^

**FIGURE 2:**
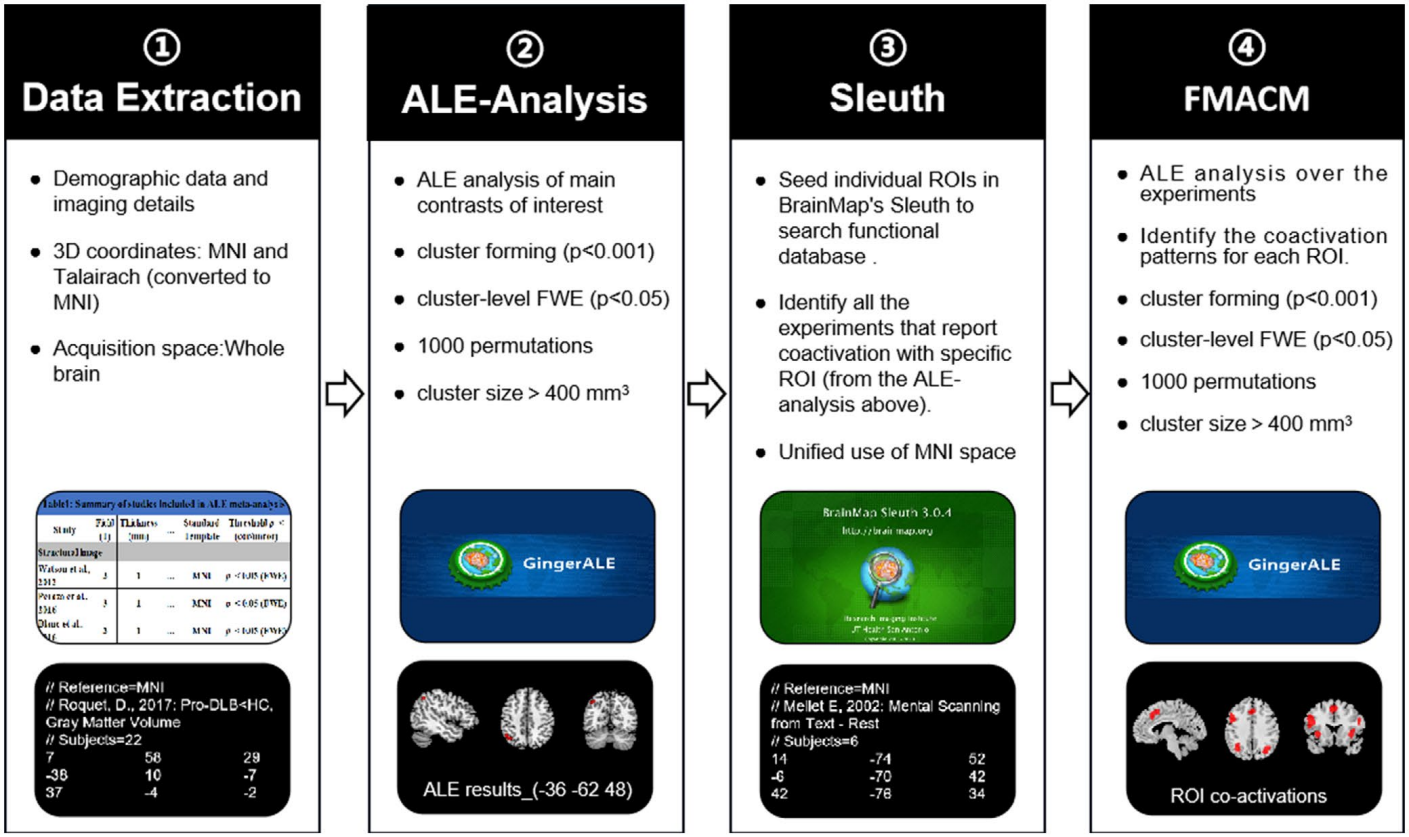
Anatomical/activation likelihood estimation (ALE) and FMACM flowchart. Pipeline showing the process of ALE and FMACM analyses and the related software, Ginger ALE and Sleuth, leading to the brain converging regions and their co‐activation regions. ① Data Extraction: Literature basic information, demographic data, experimental and imaging details and the 3D coordinates were extracted from eligible articles. ② ALE analysis: The main contrasts of interest were performed ALE analysis in MNI space using the Ginger ALE software, leading to the brain converging regions. ③ Sleuth: Create spherical ROIs of nodes using peak foci coordinates of the corrected results from ALE analysis. Then, seed individual ROIs in BrainMap's Sleuth to search functional database. Use MNI brain space. ④ FMACM Analysis: Ginger ALE software was used to perform FMACM analysis with appropriate and consistent thresholds to identify ALE meta‐analysis–co‐activated brain regions

A flowchart providing a visual interpretation of the data extraction, ALE meta‐analysis, and FMACM analysis is shown in Figure 2.

### 2.7 FMACM analysis

The fMACM analysis used data derived from the BrainMap database (screened on April 16, 2021).^33^, ^52^ The key idea of fMACM is to identify co‐activation patterns of each specific ROI.^53^ In our fMACM, each significant ROI is derived from the above ALE meta‐analysis. All experiments in the BrainMap database that reported group analyses of task‐based activations of healthy subjects were first identified, and which featured at least one focus of neural activation in the respective seed. According to this, the ALE meta‐analysis over the experiments was carried out yielding the whole‐brain co‐activation patterns for each ROI. The significance was evaluated using 1000 permutations, with a cluster‐forming threshold of *p *< 0.001, and corrected with a cluster‐level FWE threshold of *p *< 0.05.^54^

## 3 RESULTS

### 3.1 Study inclusion and characteristics

The literature search identified 8,615 potential publications. The number of studies was reduced using a four‐step assessment, such as literature identification, literature screening, eligibility assessment, and study inclusion. After the removal of the duplicates, 7,187 publications remained. A total of 4,641 publications of non‐original studies were excluded based on article categories, titles, and abstracts (ie, books/book sections (k = 3,280), reviews/meta‐analysis (k = 878), trials/protocols (k = 60), commentaries/editorials/letters (k = 41), guidelines (k = 12), case reports (k = 271), meeting abstracts (k = 99), and irrelevant studies (ie, not imaging study, no contrast between DLB and HCs/AD) (k = 2,374) were excluded, resulting in 172 publications). After full‐text screening, 146 articles were excluded due to incompatible selection criteria with our study. Finally, 26 eligible studies for ALE analysis were identified according to the search criteria mentioned above. The eligible articles included a total of 12 VBM studies, 10 FDG‐PET studies, and 5 SPECT studies (Figure 1; see Figure S1 for details). One study employed simultaneously PET and VBM.^55^

The general information of the eligible studies, image data acquisition equipment and parameters, statistical threshold, standard space, and quality scores is summarized in Table 1. The study information including sample size, demographic characteristics of the subjects, evaluation of the cognitive function, movement disorder, and diagnostic criteria is reported in Table 2.

**TABLE 2:** Demographic characteristics of the included studies

N	Study	Sample (male)	Age (years ± SD)	Disease Duration (±SD)	Education (years)	MMSE*/MoCA	UPDRS III	H‐Y	LEDD (mg)	Diagnostic criteria
Structural image										
1	Burton et al., 2002^11^	DLB:25 (18); AD:30 (14); HC:25 (13)	DLB (75.4 ± 6.8); AD (78.1 ± 5.3); HC (76.2 ± 4.7)	(Disease, mo) DLB (38.4 ± 18.3); AD (43.5 ± 26.1); HC (NA)	NA	DLB (13.3 ± 7.6)*; AD (16.4 ± 4.3)*; HC (28.1 ± 1.5)	NA	NA	NA	DLB (McKeith et al., 1996); AD (McKhann et al., 1984)
2	Brenneis et al., 2004^127^	DLB:10 (6); AD:10 (3); HC:10 (6)	DLB (70.0 ± 5.6); AD (73.1 ± 7.6); HC (65.1 ± 8.1)	NA	NA	DLB (21.2 ± 3.9)*; AD (17.4 ± 7.9)*; HC (28.8 ± 1.6)	NA	NA	NA	DLB (McKeith et al., 1996); AD (McKhann et al., 1984)
3	Ishii et al., 2007^55^	mild DLB:20 (9); mild AD:20 (7); HC:20 (5)	DLB (74.5 ± 4.9); AD (74.1 ± 3.3); HC (72.9 ± 3.3)	NA	NA	mild DLB (24.0 ± 2.2)*; mild AD (24.0 ± 2.2)*; HC (29.8 ± 0.6)	NA	NA	NA	DLB (McKeith et al., 1996); AD (McKhann et al., 1984)
4	Sanchez‐Castaneda et al., 2009^128^	DLB:12 (8); HC:16 (8)	DLB (71.1 ± 10.8); HC (71.8 ± 7.6)	(Parkinsonism, mo) DLB (32.6 ± 16.1); HC (NA)	DLB (11 ± 6); HC (7.7 ± 6.5)	DLB (19 ± 6.2)*; HC (28.6 ± 2)	DLB (27.3 ± 11); HC (NA)	DLB (2.8 ± 0.6); HC (NA)	DLB (471.4 ± 439.5); HC (NA)	DLB (McKeith et al., 2005);
5	Takahashi et al., 2010^129^	DLB:43 (17); AD:51 (20); HC:40 (20)	DLB (72.7 ± 4.5); AD (72.6 ± 2.9); HC (72.0 ± 3.8)	NA	NA	DLB (19.0 ± 3.5)*; AD (18.7 ± 4.0)*; HC (29.6 ± 0.8)	NA	NA	NA	DLB (McKeith et al., 1996); AD (McKhann et al., 1984)
6	Watson et al., 2012^12^	DLB:35 (8); AD:36 (15); HC:35 (15)	DLB (78.4 ± 6.9); AD (78.3 ± 5.8); HC (76.7 ± 5.2)	(Dementia, mo) DLB (41 ± 21)*; AD (53 ± 27)	DLB (10.8 ± 2.6); AD (11.1 ± 3.5); HC (11.7 ± 2.6)	DLB (20.3 ± 5.3)*; AD (19.5 ± 4.4)*; HC (29.1 ± 1.0)	DLB (26.0 ± 10.7)*^†^; AD (5.4 ± 4.3); HC (2.0 ± 1.9)	NA	NA	DLB (McKeith et al., 1996, 2005); AD (McKhann et al., 1984)
7	Borroni et al., 2015^130^	DLB:13 (7); HC:10 (3);	DLB (74.2 ± 5.2); HC (62.2 ± 8.0);	(Diagnosis, years) DLB (4.2 ± 2.6); HC (NA);	HC (8.2 ± 3.5); DLB (6.3 ± 3.5);	DLB (20.31 ± 6.05); HC (NA);	DLB (20.1 ± 8.6)*; HC (0.0);	NA	DLB (279.6 ± 224.6); HC (NA);	DLB (McKeith et al., 2005);
8	Blanc et al., 2016^131^	Pro‐DLB:28 (12); Pro‐AD:27 (20); HC:33 (15)	Pro‐DLB (67.5 ± 9.2); Pro‐AD (69.3 ± 7.8); HC (72.4 ± 10.4)	NA	NA	Pro‐DLB (27.6 ± 2.1);* Pro‐AD (26.9 ± 1.9)*; HC (29.4 ± 0.9)	NA	NA	NA	pro‐AD (Dubois et al., 2007); Pro‐DLB (McKeith et al., 2005) (Petersen et al., 2004) (Donaghy et al., 2014)
9	Heitz et al., 2016^132^	DLB:33 (16); AD:15 (8); HC:16 (7)	DLB (68 ± 8.4); AD (70.9 ± 11.1); HC (68.3 ± 10.5)	(Disease, year) DLB (4.6 ± 4.2); AD (3.6 ± 1.8); HC (NA)	DLB (12.4 ± 3.2); AD (13.5 ± 3.6); HC (11.9 ± 3.2)	DLB (27.2 ± 1.8)*; AD (27 ± 2.6)*; HC (29.3 ± 0.9)	NA	NA	NA	DLB (McKeith et al., 2005); AD (Dubois B, 2007)
10	Peraza et al., 2016^133^	DLB:19 (13); AD:18 (15); HC:16 (13);	DLB (76.32 ± 6.45); AD (75.39 ± 8.6); HC (76.75 ± 5.93)	(Diagnosis, years) DLB (1.0 ± 0.6)^†^; AD (1.65 ± 0.8); HC (NA)	NA	DLB (23.05 ± 4.13)*; AD (21.83 ± 3.8)*; HC (29.1 ± 0.88);	DLB (14.95 ± 5.47)*^†^; AD (1.56 ± 1.68); HC (1.44 ± 1.93)	NA	NA	DLB (McKeith et al., 2005); AD (McKhann et al., 1984)
11	Roquet et al., 2017^134^	Pro‐DLB:54 (23); mild DLB:15 (8); Pro‐AD:16 (11); mild AD:28 (9); HC:22 (10)	Pro‐DLB:(69.3 ± 9.0); mild DLB:(74.3 ± 10.4)*; Pro‐AD:(75.3 ± 9.2)*; mild AD:(74.1 ± 8.8); HC:(65.6 ± 9.2)	NA	NA	Pro‐DLB: (27.6 ± 1.4)*; Mild DLB:(20.7 ± 3.4)*; Pro‐AD:(27.1 ± 1.4)*; Mild AD:(19.3 ± 3.3)*; HC:(29.0 ± 1.0)	NA	NA	NA	pro‐AD (Dubois et al., 2007); DLB (McKeith et al., 2005); Pro‐DLB (McKeith et al., 2005) (Petersen et al., 2004).
12	Nemoto et al., 2021^135^	DLB:101 (51); AD:69 (33); HC:38 (10)	DLB (73.25 ± 8.05); AD (71.58 ± 6.33); HC (71.03 ± 6.28)	NA	NA	DLB (22.21 ± 4.86)*; AD (21.32 ± 3.95)*; HC (28.21 ± 1.26)	NA	NA	NA	DLB (DSM−5); AD (DSM−5)
Functional imaging										
PET										
1	Imamura et al., 1997^109^	DLB:19 (5); AD:19 (5)	DLB (72.6 ± 4.8); AD (72.8 ± 5.6)	(Cognitive, mo): DLB (24.2 ± 13.7); AD (24.1 ± 13.8)	NA	DLB (17.7 ± 4.1); AD (18.4 ± 4.1)	NA	NA	NA	DLB (McKeith et al., 1996); AD (McKhann et al., 1984)
2	Ishii et al., 2007^55^	mild DLB:20 (9); mild AD:20 (7); HC:20 (5)	DLB (74.5 ± 4.9); AD (74.1 ± 3.3); HC (72.9 ± 3.3)	NA	NA	mild DLB (24.0 ± 2.2)*; mild AD (24.0 ± 2.2)*; HC (29.8 ± 0.6)	NA	NA	NA	DLB (McKeith et al., 1996); AD (McKhann et al., 1984)
3	Perneczky et al., 2007^136^	DLB:21 (11); HC:16 (7)	DLB (71.1 ± 4.4); HC (67.88 ± 10.0)	(Disease, years): DLB (3.4 ± 2.1); HC (NA)	DLB (10.4 ± 2.3); HC (11.69 ± 4.0)	DLB (20.8 ± 4.8)*; HC (30 ± 0.0)	DLB (30.4 ± 15.6); HC (NA)	NA	NA	DLB (McKeith et al., 1996); AD (McKhann et al., 1984)
4	Yong et al., 2007^137^	DLB:7 (3); HC:15 (6);	DLB (74.3 ± 6.9)*; HC (65.3 ± 5.6);	(Disease, years) DLB (2.0 ± 0.8); HC (NA); (Dementia, years) DLB (1.9 ± 0.7); HC (NA)	NA	DLB (27.3 ± 2.1); HC (NA);	NA	DLB (2.1 ± 1.2); HC (NA);	NA	DLB (McKeith et al., 2005);
5	Teune et al., 2010^138^	DLB:6 (NA); HC:18 (NA)	DLB (71 ± 7)*; HC (56 ± 14)	(Disease, years) DLB (3 ± 2); HC (NA)	NA	NA	NA	NA	NA	DLB (McKeith et al., 2005)
6	Iizuka et al., 2016^111^	DLB:24 (NA); AD:24 (NA)	Medians (interquartile ranges) DLB (73 (68, 79)); AD (74 (69, 81))	(Disease, year, Medians (interquartile ranges)) DLB (2.8 (1.8,3.2)); AD (2.3 (1.6,2.6))	Medians (interquartile ranges) DLB (16 (12,18)); AD (15 (12,18))	Medians(interquartile ranges) DLB (23 (20.5,24)); AD (23 (21,24.5))	NA	NA	NA	DLB (McKeith et al., 2005); AD (McKhann et al., 1984)
7	Iizuka et al., 2017^139^	DLB:34 (18); HC:18 (9)	DLB (76.9 ± 2.3); HC (77.1 ± 1.3)	NA	DLB (13.4 ± 1.9); HC (12.8 ± 1.3)	DLB (23.6 ± 2.3)*; HC (29.3 ± 0.5)	NA	NA	NA	DLB (McKeith et al., 2005)
8	Liu et al., 2017^140^	DLB:37 (21); HC:5 (NA)	DLB (71.8 ± 9.1); HC (NA)	NA	DLB (10.3 ± 4.4); HC (NA)	MMSE: DLB (16.6 ± 7.4); HC (NA); MoCA: DLB (9.6 ± 7.0); HC (NA)	DLB (13.9 ± 12.4); HC (NA)	NA	NA	DLB (McKeith et al., 2005)
9	Liguori et al., 2019^141^	DLB:10 (8); HC:35 (19)	DLB (69.02 ± 7.71); HC (67.89 ± 4.95)	(Disease, year) DLB (2.15 ± 1.26); HC (NA)	NA	DLB (23.6 ± 5.20)*; HC (29.40 ± 1.22)	DLB (15.01 ± 6.45); HC (NA)	NA	NA	DLB (McKeith et al., 2017)
10	Iizuka et al., 2020^142^	DLB:50 (26); AD:50 (24); HC:50 (25)	DLB (76.9 ± 5.0); AD (76.3 ± 5.3); HC (77.3 ± 5.4)	NA	DLB (14.5 ± 2.4); AD (14.3 ± 2.4); HC (14.1 ± 2.5)	DLB (22.0 ± 1.4)*; AD (21.7 ± 1.8)*; HC (29.6 ± 0.5)	NA	NA	NA	DLB (McKeith et al., 2017); AD (McKhann et al., 2011)
SPECT										
1	Colloby et al., 2002^94^	DLB:23 (9); AD:48 (21); HC:20 (9)	DLB (75.9 ± 8.6); AD (77.9 ± 7.0); HC (75.4 ± 5.1)	NA	NA	DLB (16.0 ± 6.1)*; AD (17.4 ± 5.5)*; HC (28.5 ± 1.5)	NA	NA	NA	DLB (McKeith et al., 1996); AD (McKhann et al., 1984)
2	Firbank et al., 2003^58^	DLB:15 (8); HC:37 (20)	DLB (76.1 ± 7.7); HC (75.0 ± 6.7)	(Disease, mo, median (range)): DLB (23 (2–48)); HC (NA); (Dementia, mo): DLB (26 ± 16); HC (NA)	DLB (15.2 ± 0.6); HC (16.7 ± 2.5)	DLB (18.1 ± 5.1)*; HC (28.1 ± 1.5)	DLB (26 ± 17); HC (NA)	NA	NA	DLB (McKeith et al.,1996); AD (McKhann et al., 1984)
3	Takahashi et al., 2010^143^	DLB:44 (DLB‐P:13 (7); DLB‐nP:31 (15)); HC:16 (NA)	DLB‐P (80.3 ± 4.4); DLB‐nP (78.0 ± 7.1)	NA	NA	DLB‐P (18.2 ± 3.5); DLB‐nP (19.9 ± 5.6)	NA	NA	NA	DLB (McKeith et al., 1996)
4	Misch et al., 2014^144^	DLB:30 (20); HC:30 (20)	DLB (72.3 ± 1.7); HC (73.1 ± 1.2)	(Disease, year) DLB (3.7 ± 0.4); HC (NA)	DLB (14.6 ± 0.7); HC (15.17 ± 0.6)	NA	NA	NA	NA	DLB (McKeith et al., 2005)
5	Park et al., 2018^82^	DLB:33 (18)*; HC:30 (7)	DLB (74.1 ± 4.9)*; HC (68.5 ± 3.6)	(Disease, mo) DLB (24.2 ± 18.0); HC (NA)	DLB (7.6 ± 4.5); HC (7.1 ± 4.8)	DLB (19.8 ± 4.7); HC (NA)	NA	NA	NA	DLB (McKeith et al., 2005)

Abbreviations: AD, Alzheimer's disease; DLB, dementia with Lewy bodies; DLB‐nP, DLB patients without parkinsonism; DLB‐P, dementia with Lewy bodies with parkinsonism; HC, healthy controls; H‐Y, Hoehn‐Yahr; LEDD, levodopa equivalent dose; MMSE, Mini‐Mental State Examination; mo, month; MoCA, Montreal Cognitive Assessment; N, study number; NA, data not available; pro‐AD, prodromal Alzheimer's disease; pro‐DLB, prodromal dementia with Lewy bodies; SD, standard deviation; UPDRS III, Unified Parkinson's Disease Rating Scale, motor subscale.

*p < 0.05, compared with HC; ^†^ *p* < 0.05, compared with AD.

### 3.2 Anatomical/activation likelihood estimation meta‐analysis results

#### 3.2.1 Regions with structural changes between DLB and HCs

**FIGURE 3:**
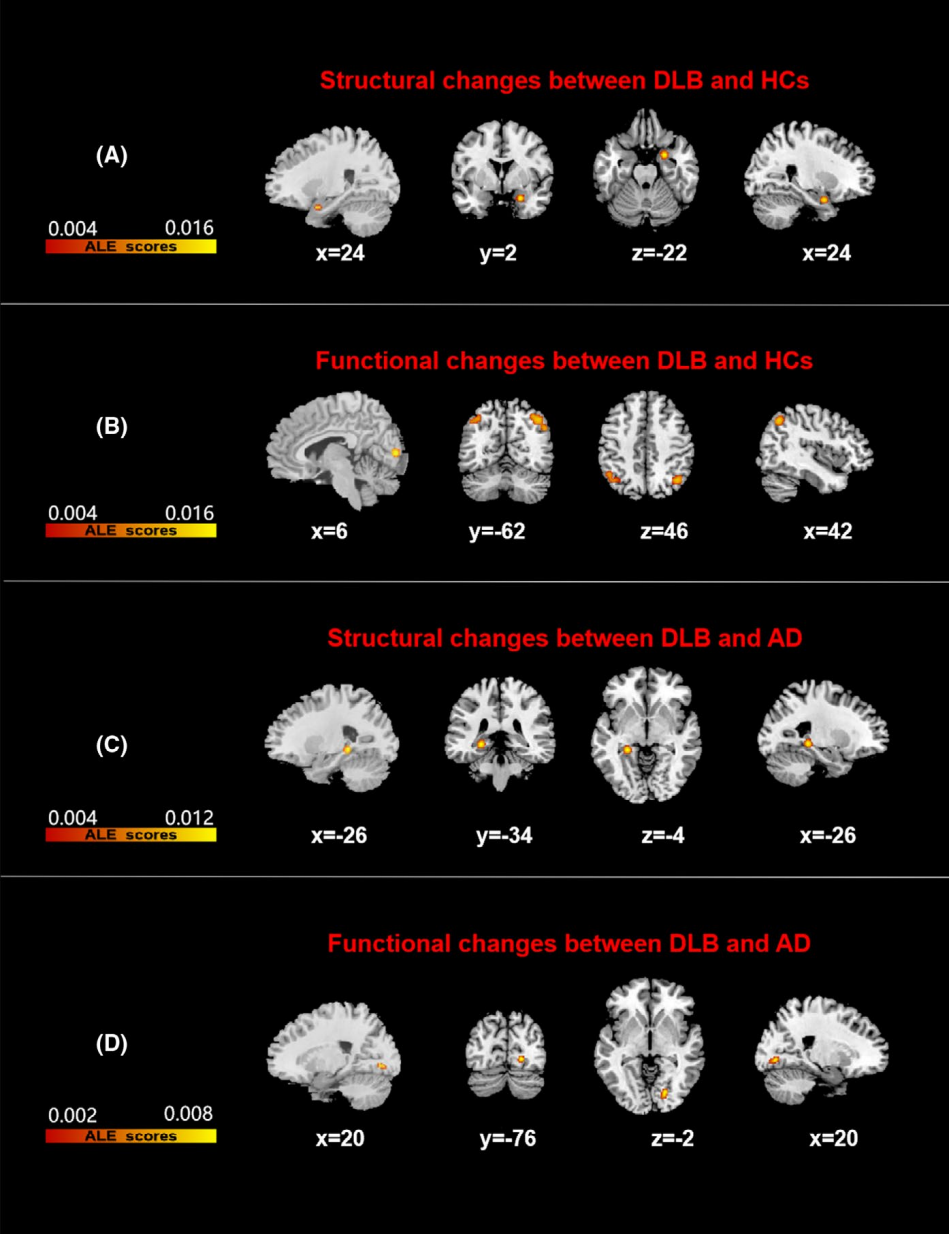
Results of all Anatomical/activation likelihood estimation (ALE) analyses. Figures B and C were corrected by FWE at cluster level, with a cluster‐forming threshold of *p* < 0.001 and cluster‐level inference of 0.05. Figures A and D were uncorrected, *p* < 0.001. Results were superimposed on a brain template using MRIcron software in MNI space. The left side of the image represents the left hemisphere of the brain. Color bars represent anatomical/activation likelihood estimation scores. DLB, dementia with Lewy body, HCs, healthy controls, AD, Alzheimer disease

Based on the structural analysis of DLB <HCs, no converging brain area was found after FWE correction. Atrophy of the right parahippocampal gyrus tended to converge in DLB patients (uncorrected, *p* < 0.001; Figure 3A, Table 3).

**TABLE 3:** All clusters from ALE analysis

Cluster No.	Volume (mm^3^)	MNI			Anatomical regions	Maximum ALE value	*p* value	Fail‐Safe N (%)^b^
		x	y	z				
Structural imaging analysis based on DLB <HCs								
1	472	24	2	−22	Right parahippocampal gyrus	0.01760611	0.00000024^a^	–
Functional imaging analysis based on DLB <HCs								
1	1,824	42	−62	46	Right inferior parietal lobule	0.01439173	0.00000607	>30%
2	696	6	−86	6	Right lingual gyrus	0.01811388	0.00000019	30% > FSN > 20%
3	584	−36	−62	48	Left inferior parietal lobule	0.01098338	0.00009512	20% > FSN > 10%
Structural imaging analysis based on AD <DLB								
1	568	−24	−36	−4	Left parahippocampal gyrus	0.01580051	0.00000023	>30%
Functional imaging analysis based on DLB <AD								
1	360	20	−76	−2	Right lingual gyrus	0.00954966	0.00000309^a^	–

Note

–indicates no FSN evaluation.

Abbreviations: AD, Alzheimer's disease; DLB, dementia with Lewy bodies.

^a^ Was the result that cannot stand the FWE correction (uncorrected, *p *< 0.001).

^b^ Represents the ratio to the number of experiments included in the meta‐analysis.

#### 3.2.2 Regions with functional changes between DLB and HCs

The functional analysis based on DLB <HCs showed that the reduced functional activity in DLB patients was mainly located in the bilateral inferior parietal lobule and right lingual gyrus (Figure 3B, Table 3).

#### 3.2.3 Regions with structural changes between DLB and AD

The structural analysis based on AD <DLB showed that the local brain atrophy in the left medial temporal lobe (MTL) was more severe in AD patients compared with that in DLB patients. Peak cluster was found in the left parahippocampal gyrus (Figure 3C, Table 3). No enough experiments were available to analyze DLB <AD (n = 35; 3 foci; 2 experiments).

#### 3.2.4 Regions with functional changes between DLB and AD

Based on the DLB <AD functional analysis, DLB patients had a tendency of lower metabolism in the right lingual gyrus compared with that in AD patients (uncorrected, *p* < 0.001; Figure 3D, Table 3). No enough experiments were available to analyze AD <DLB (n = 93; 11 foci; 3 experiments).

### 3.3 Jackknife sensitivity analysis

In this study, jackknife sensitivity analysis was performed on the corrected ALE results. To this end, 14 and 6 different ALE meta‐analyses of "functional changes between DLB and HCs" and "structural changes between DLB and AD", respectively, were conducted. The sensitivity analysis revealed that the reduced functional activity of DLB patients in the right inferior parietal lobule was the most robust result, replicable in all the 14 datasets. The reduced functional activity in the right lingual gyrus and left inferior parietal lobule remained relatively highly replicable. This was due to the still significant value in the combination of at least 9 combinations of the datasets (Table S3). However, the less atrophy of the left parahippocampal gyrus in DLB patients compared with AD was a replicable result in only three studies (Table S4).

### 3.4 Fail‐safe N analysis

The last column of Table 3 shows the fail‐safe percentage of the additional noise that must be added to each meta‐analysis to cause the convergence failure of previously determined clusters. Overall, the FSN assessment results were consistent with the jackknife sensitivity analysis. The most stable result was a decreased functional activity of the right inferior parietal lobule in patients with DLB. The reduction of functional activity in the right lingual gyrus and the left inferior parietal lobule remained relatively highly stable. This was due to the still significant value in the combination of more than 10% noise datasets. Moreover, the less atrophy in the left parahippocampal gyrus in DLB patients compared with AD remained a significant result with the addition of 33% noise (Table 3).

### 3.5 FMACM results

#### 3.5.1 Article inclusion

The corrected result was selected as the ROI. It was represented by the bilateral inferior parietal lobule, right lingual gyrus, and the left parahippocampal gyrus. At the time of the fMACM analysis (April 16, 2021), the database consisted of 1,315,198 locations/coordinates, 76,016 unique subjects, and 16,901 experiments from 3,406 publications. Detailed descriptions of each of the four ROIs retrieved from the database are summarized in Table 4. For example, the ROI of the right inferior parietal lobule was identified in 32 experiments, with data of 526 subjects and 470 foci being subjected to further ALE analysis.

**TABLE 4:** Details of each ROI retrieved from the database

Number	ROIs name	MNI			Experiments	Subjects	Foci
		x	y	z			
1	Right inferior parietal lobule	42	−62	46	32	526	470
2	Left inferior parietal lobule	−36	−62	48	58	891	667
3	Right lingual gyrus	6	−86	6	53	724	693
4	Left parahippocampal gyrus	−24	−36	−4	27	398	353

Note

Search date: April 16, 2021.

#### 3.5.2 fMACM co‐activations

1. Co‐activation patterns of differences between DLB and HC images

**FIGURE 4:**
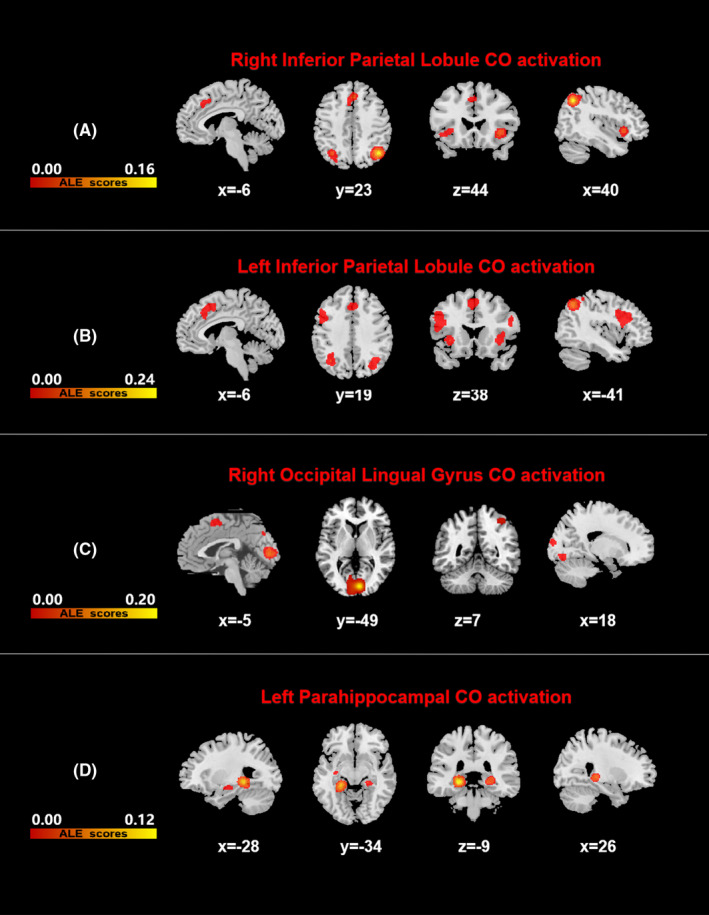
Results of all fMACM co‐activated brain areas. All results were FWE corrected with a cluster‐forming threshold of *p* < 0.001 and cluster‐level inference of 0.05. Results were superimposed on a brain template using MRIcron software in MNI space. Color bars represent anatomical/activation likelihood estimation scores. DLB, dementia with Lewy body, HCs, healthy controls, AD, Alzheimer's disease

The right inferior parietal lobule showed a co‐activation with the bilateral inferior parietal lobule, medial frontal gyrus, insula, anterior cingulate gyrus, and left precuneus. The left inferior parietal lobule was co‐activated with the bilateral inferior parietal lobule, inferior frontal gyrus, insula, anterior cingulate gyrus, left superior frontal gyrus, and right precuneus. The right lingual gyrus showed a co‐activation with the bilateral lingual gyrus, right cuneus, right fusiform gyrus, left medial frontal gyrus, and right inferior parietal lobule (Figure 4A‐C, Table 5).

**TABLE 5:** Functional meta‐analytic connectivity models (fMACM) co‐activated brain areas

Cluster No.	Volume (mm^3^)	MNI			Anatomical regions	Maximum ALE value	*p* value
		X	Y	Z			
Right inferior parietal lobule co‐activations							
1	6,528	42	−62	46	Right inferior parietal lobule	0.15539981	0.00000000
2	2,680	36	20	−2	Right insula	0.04352491	0.00000000
3	2,264	2	30	42	Right medial frontal gyrus, Right anterior cingulate gyrus	0.03657617	0.00000000
		−6	18	46	Left medial frontal gyrus, Left anterior cingulate gyrus	0.01930561	0.00003330
4	1,992	−34	20	−2	Left insula	0.03323909	0.00000000
5	2,216	−34	−62	46	Left inferior parietal lobule	0.03736221	0.00000000
		−28	−76	42	Left precuneus	0.01801638	0.00007551
Left inferior parietal lobule co‐activations							
1	8,760	−44	12	26	Left inferior frontal gyrus	0.04009932	0.00000000
2	8,584	−36	−62	48	Left inferior parietal lobule	0.23778364	0.00000000
		−18	−74	52	Left precuneus	0.02552385	0.00014917
3	7,088	40	−60	46	Right inferior parietal lobule	0.05201638	0.00000000
		36	−60	44	Right precuneus	0.05182976	0.00000000
4	5,992	−4	16	52	Left superior frontal gyrus	0.04075132	0.00000000
		−2	28	40	Left anterior cingulate gyrus	0.03539044	0.00000000
		2	28	40	Right anterior cingulate gyrus	0.03512712	0.00000001
5	1,968	−34	22	−2	Left insula	0.04484950	0.00000000
6	1,760	36	22	−2	Right insula	0.03512499	0.00000007
7	1,112	52	16	28	Right inferior frontal gyrus	0.02704643	0.00000134
Right lingual gyrus co‐activations							
1	17,904	6	−86	6	Right lingual gyrus	0.20958409	0.00000000
		18	−96	12	Right cuneus	0.02316909	0.00000977
		26	−70	−8	Right fusiform gyrus	0.01793012	0.00024772
2	2,464	−2	6	58	Left medial frontal gyrus	0.03356890	0.00000001
3	920	−30	−84	−8	Left middle occipital gyrus	0.02497809	0.00000302
4	656	−26	−86	20	Left middle occipital gyrus	0.02061020	0.00004895
		−22	−92	10	Left lingual gyrus	0.01845707	0.00018086
5	656	40	−48	58	Right inferior parietal lobule	0.02217278	0.00001846
6	632	4	−76	38	Right cuneus	0.02486873	0.00000324
Left parahippocampal gyrus co‐activations							
1	5,512	−24	−36	−4	Left parahippocampal Gyrus Left hippocampus, Left thalamus	0.10880894	0.00000000
		−34	−50	−12	Left fusiform gyrus	0.01375041	0.00035655
2	2,016	26	−30	−2	Right thalamus	0.03932637	0.00000000
3	1,608	−22	−4	−18	Left parahippocampal gyrus	0.01816290	0.00001711
4	648	32	−4	−16	Right parahippocampal gyrus	0.01670547	0.00004697

2. Co‐activation patterns of differences between DLB and AD images

The co‐activation brain regions of the left parahippocampal gyrus consisted of the bilateral parahippocampal gyrus, thalamus, and left hippocampus (Figure 4D, Table 5).

## 4 DISCUSSION

This ALE meta‐analysis is the first quantification of the location of cerebral changes across different imaging modalities in DLB. In addition, it is the first application of fMACM to characterize co‐activated neural networks associated with the damaged brain areas in DLB. This study found that the right parahippocampal gyrus atrophy in DLB patients tended to converge. Moreover, the functional activity of the bilateral parietal lobe and right occipital lobe significantly decreased compared with those in HC patients. Structural differences between DLB and AD were preferentially concentrated in the left parahippocampal gyrus, and functional differences tended to converge to the right lingual gyrus. Furthermore, these convergent brain regions co‐activated with extensive brain regions, covering multiple neural networks. These local convergent brain regions might be potential image markers of DLB damage or differentiation from AD. Moreover, they might be key "nodes" in those co‐activated neural networks, forming the basis of the network degradation hypothesis.

### 4.1 Local changes and co‐activation patterns of the differences between DLB and HCs

DLB patients showed a hypometabolism of the parietal and occipital lobes, but no convergent structural difference compared with HC patients. This might suggest that brain function abnormalities in DLB patients potentially occur before the structural atrophy. Functional modalities can detect the early stage of brain dysfunction before the morphological changes with high sensitivity.^56^

Visual perception is a complex and active process, which depends on the working memory of the visual space, especially through the ocular exploration of the visual scene to realize the spatiotemporal integration of the perceived elements. Neuropsychological data suggest that the right inferior parietal lobule may be the neural substrates of the spatiotemporal integration.^57^ Therefore, the reduced functional activity of the inferior parietal lobule may be related to the visuospatial perception deficits present in persons with DLB.^58^ The reduced occipital activity is one of the diagnostic biomarkers.^1^ Some researchers suggested that the occipital hypoperfusion is associated with visual hallucinations.^59^ Others reported that it is associated with cognitive fluctuation and global cognitive function.^60^, ^61^ The pathological process of widespread spongiform changes and gliosis in the long projection fibers may at least partly contribute to the characteristic imaging features of DLB.^22^ Occipital hypometabolism accurately classifies coincident DLB (80% sensitivity and 100% specificity).^62^ These results provide a basis for the rational use of the parietal‐occipital lobe activity as an imaging marker for the diagnosis of DLB.

Bilateral inferior parietal lobule–co‐activated bilateral frontal and parietal lobule–related brain regions, together forming the bilateral FPN, are typically associated with attentional and executive functions.^63^ The decreased FC of the FPN was associated with the severity and frequency of cognitive fluctuations in DLB patients.^28^ Furthermore, the co‐activation of the bilateral inferior parietal lobule was found in the bilateral insula and in the anterior cingulate gyrus, forming the classical salience network (SN).^64^ SN is responsible for the evaluation of the surrounding information, and socio‐emotional and visceral autonomic processing,^65^ with abnormalities being described in a variety of psychiatric disorders.^66^, ^67^ Poor connectivity in this network in DLB patients can cause mood disturbances, which are common in DLB.^27^ The right lingual gyrus showed a co‐activation with the bilateral lingual gyrus. These regions belong to the VIS,^64^ which is characterized by a common activation during visuospatial creativity tasks.^68^, ^69^ In addition, it is related to a novelty detection processing, construction of novel images, and mental imagery.^70^, ^71^ The co‐activation of the right lingual gyrus also included medial prefrontal lobe related to DMN and parietal cortex related to FPN. Indeed, in the posterior parietal region, it is possible to anatomically distinguish the spatial representation process based on the integration of space and time (visuospatial working memory) and the immediate process of selecting the important visual information to be maintained (attention).^57^ This feature might imply the possibility that the parietal lobe could serve as a hub for coordinating multiple network functions. These findings suggested that the co‐activation patterns of these regions could be attributed to some recognized neural networks. However, the fact that the neuromodulation of these neural networks can improve the cognitive and mental disorders in DLB group is yet to be explored.

### 4.2 Local changes and co‐activation patterns of the differences between DLB and AD

The left parahippocampal atrophy was less in DLB patients than in AD patients, supporting the idea that the MTL of DLB was relatively preserved. The parahippocampal gyrus is responsible for high‐level neurological activities such as emotion, learning, and memory. It is also an important structure to ensure the normal hippocampal function. Its structural damage may cause abnormal emotional and cognitive behaviors. The volume of the MTL structure such as the parahippocampal gyrus was significantly reduced in AD patients due to the large amount of AD‐type pathological deposition.^72^ The loss of MTL gray matter is associated with memory impairment, even at a prodromal stage.^73^ ALE meta‐analysis studies reveal that AD structurally affects the (trans‐) entorhinal, hippocampal regions and the amygdala,^74^, ^75^ compared with HCs. These findings, combined with our results, provide richer evidence that MTL volume could serve as an image marker to distinguish DLB from AD. In addition, pathological studies reported the existence of a relative preservation of the hippocampus in patients with AD. However, it is often associated with non‐amnestic clinical manifestations, in which cortical atrophy is the main feature, whereas the MTL is relatively well preserved.^76^, ^77^ Therefore, our hypothesis was that the relative preservation of MTL in DLB patients might be associated with the relative preservation of the memory.^78^ Our results from the perspective of quantitative meta‐analysis demonstrated that DLB and AD patients have different patterns of brain atrophy. This aspect supports the use of the MTL volume as a biomarker to distinguish the two. Of note, the findings of our analysis likely underestimate the extent and severity of cerebral changes in DLB because the small number of whole‐brain results in a reduced power are not enough to detect significant voxels.

Dementia with Lewy bodies patients had a tendency of having a lower metabolism in the right lingual gyrus compared with to that in AD patients, as often reported in previous studies.^22^, ^79^ The lingual gyrus, located in the visual region 2 (V2), is closely connected to visual region 1 (V1). Additionally, the lingual gyrus is a crucial component of the dorsal visual pathway for visual processing and spatial memory.^80^ Therefore, our hypothesis was that the lower metabolism of the lingual gyrus in DLB patients might be related to the more common visual hallucinations and visuospatial disorders. Indeed, the reduced occipital activity (hypoperfusion or hypometabolism) including the lingual gyrus, found by SPECT or FDG‐PET, is considered a supportive imaging biomarker for DLB.^1^ FDG‐PET occipital hypometabolism correlates with visual cortex neuropathology in DLB.^22^ In addition, an autopsy‐confirmed study suggested that the above correlation could distinguish DLB from AD with high accuracy.^81^ The ALE meta‐analysis of AD functional images showed that the hypoperfusion and hypometabolism in the parietal lobe (angular gyrus, supramarginal gyrus, and precuneus)^74^ and posterior cingulate gyrus^75^ were convergent compared with HCs. Our study found that patients with DLB had functionally affected bilateral inferior parietal lobules and right lingual gyrus. Overall, these findings further demonstrated that decreased occipital activity is more frequently seen in DLB, while decreased temporal parietal activity is common in both AD and DLB.^79^ This allowed the distinction between DLB and AD with a sensitivity of 90% and a specificity of 80%.^81^ Furthermore, hypoperfusion in the right lingual gyrus precedes the hypoperfusion in the frontal and temporal cortices, underlining the changes in the early stages of the disease.^82^ These aspects suggest that the measurement of the occipital metabolism/perfusion, even in the early stages of the disease, might be an informative diagnostic aid to distinguish DLB from AD. Thus, the combination of hippocampal volumes and occipital activity allows the distinction of DLB patients from those with AD with a higher level of accuracy.^83^

The co‐activated brain areas of the left parahippocampal gyrus involve the bilateral parahippocampal gyrus, hippocampus, and thalamus, which are mainly located in the DMN.^64^ The DMN has an important role in several cognitive functions and includes the prefrontal cortex, bilateral parahippocampal gyrus, hippocampus, thalamus, inferior‐lateral‐parietal lobule, and precuneus.^84^ Reduced DMN connectivity is associated with decreased memory performance, slower processing speed, and decreased executive function.^85^, ^86^, ^87^ Alterations in the DMN are involved in a range of neurodegenerative disorders such as AD, Parkinson's disease, and frontotemporal dementia.^88^, ^89^, ^90^, ^91^ This pattern of co‐activation of DMN‐related brain regions driven by the ROI with greater atrophy in AD compared with DLB highlights a possible structural basis for the abnormal reduction of the DMN resting state activity in AD patients. The DMN is not hypoactive in DLB patients, with increased FC concentrated in the posterior part of the DMN.^31^, ^92^ This is consistent with the idea that DMN is relatively well preserved in DLB. Therefore, the integrity of DMN might provide a new perspective for the differential diagnosis between AD and DLB. However, the different role of the neuromodulation of DMN (such as transcranial magnetic stimulation) in the cognitive improvement of patients with AD and DLB needs further investigation.

### 4.3 Novel imaging modalities in DLB patients

#### 4.3.1 Molecular imaging

DAT imaging with the radioactive tracer 123I‐FP‐CIT SPECT (DaTSCAN) or 18F‐FP‐CIT PET has become a useful tool for assessing dopaminergic function in vivo. Decreased DAT transporter uptake in basal ganglia is considered to be an indicative biomarker for DLB diagnosis.^93^ In one series, 123I‐FP‐CIT SPECT discriminated pathologically proven DLB from AD with 88% sensitivity and 100% specificity as the latter is not associated with loss of striatal DAT binding.^94^ When applied to post‐mortem confirmed DLB cases, the diagnostic accuracy is higher.^95^, ^96^, ^97^ Although DAT scans are normal in approximately 20% of DLB patients (mixed DLB+AD and DLB alone), abnormal DAT scans strongly support the diagnosis of DLB.^98^ DAT scan is the best neuroimaging technique for differentiating DLB from AD, even in the early stage of the disease.^99^

123I‐metaiodobenzylguanidine (123I‐MIBG) cardiac scintigraphy is currently widely utilized in Lewy body diseases, including DLB and PD, and in REM sleep‐related behavioral disorder. [123I] MIBG cardiac scintigraphy is used to evaluate cardiac postganglionic sympathetic degeneration, which has similar sensitivity and specificity to DAT imaging.^100^, ^101^ A multicenter analysis demonstrated that an abnormal uptake has a sensitivity of 77% and a specificity of 97% for differentiating DLB from AD at 3‐year follow‐up.^102^ It can not only exclude AD but also predict the transformation from possible DLB to likely DLB,^103^, ^104^, ^105^ but it may show false positive in cases of heart failure, ischemic heart disease, etc., requiring caution in interpretation.^106^ Positive studies have been reported in premotor DLB with reduced uptake manifesting prior to reduced DAT uptake, indicating that 123I‐MIBG scintigraphy may have an even greater role in early disease.^107^ 123I‐MIBG scintigraphy was given an increased diagnostic weighting in the 2017 DLB consortium and is now considered an indicative biomarker.

In some cases, DLB pathology is characterized by amyloid protein (Aβ) and tau deposition in addition to α‐synuclein aggregation.^108^, ^109^ Studies have shown significant increase in Aβ load in more than 80% of DLB patients.^110^ The degree and distribution of Aβ deposition in DLB were similar to AD, mainly showing increased deposition in frontal lobe, precuneus, posterior cingulate gyrus, temporoparietal area, and striatum.^109^ Aβ deposition does not differentiate DLB from AD, but can be used to distinguish DLB from Parkinson's disease.^111^ Studies based on the Tau protein radioactive ligand [18F] AV‐1451 showed that compared with HC, the uptake of [18F] AV‐1451 in DLB patients increased, especially in the inferior temporal gyrus and precuneus cortex,^112^ and posterior temporal parietal and occipital cortex.^113^ Compared with DLB, the uptake range of [18F] AV‐1451 in AD patients is wider and heavier.^113^ In addition, AD showed the highest intake of the medial temporal lobe and DLB showed the lowest intake; thus, DLB and AD can be completely distinguished.^114^ Pathological α‐syn exists in many forms and is deposited in large quantities in other misfolded proteins, such as Aβ and Tau.^115^ However, several potential compounds still have low affinity for α‐syn^116^ and slow clearance.^117^ There have been no clinical trials of alpha‐syn imaging in DLB patients due to high permeability in the brain, rapid clearance, and α‐syn high selection, and high‐affinity radioactive ligand is an unmet demand.

In general, novel molecular imaging modalities are important methods for evaluating neurobiology in vivo. Radionuclides are rare tools for tracking neurotransmitters, synaptic pathology, and misfolded protein aggregation. Molecular imaging of Aβ, Tau, and α‐syn enables precise pathological quantification and may lead to innovative therapeutic opportunities.

#### 4.3.2 Dynamic functional connectivity

Functional magnetic resonance imaging (fMRI) can sensitively detect spontaneous neural activity by measuring changes in signals based on blood oxygen level–dependent imaging. Functional connectivity (FC) quantifies temporal correlations of functional activation in different brain regions, revealing specific networks,^118^ and is considered to be an important biomarker. Considering the dynamic nature of brain activity, dynamic functional connectivity (DFC) provides a new approach. It can identify and analyze temporal fluctuations of FC between brain regions on a faster timescale.^119^ Given the transient and repetitive nature of some of the key features of DLB, namely cognitive fluctuations and hallucinations, DFC studies are expected to provide new insights into the neuropathological mechanisms of the disease. However, so far, most studies on DFC evaluation of neurodegenerative diseases have focused on AD and PD, and the exploration of DFC in resting state fMRI of DLB patients is very limited. Classical static FC studies have shown reduced FC in the extensive brain network of DLB subjects, and dyssynchrony of cortical and subcortical regions is associated with cognitive fluctuations.^120^ In fact, one study, from a modeling perspective, detected significant differences in DFC in vision‐related networks (ie, occipito‐parietal lobe‐frontal and medial occipito‐frontal) and attention network (ie, right fronto‐parietal control networks) in DLB patients compared with HC, suggesting that the interdependence between networks is reduced. These temporarily disconnected networks may be related to the pathogenesis of DLB.^121^ Previous work of our research group found that DLB's dynamic functional connectivity variability and time allocation of clustering state sequences changed, which may lead to complex brain network dynamics disorder, and may make the brain lack integration and flexibility, resulting in ineffective brain function.^122^ Overall, DFC is a promising approach to better understand the neurodegenerative process of DLB and to investigate new biomarkers for disease diagnosis and prognosis. At present, studies on DFC in patients with DLB are limited and it is difficult to draw consistent conclusions.

This report has some limitations. First, the heterogeneity of the study characteristics, including different data acquisition, preprocessing protocols, statistical methods, and threshold settings, could not be entirely ruled out. Second, the number of experiments included in each analysis was small. The coordinate‐based meta‐analysis was limited to the primary studies that convey all information in the format required for statistical processing. This means that the included literature was not comprehensive. However, the quantitative meta‐analysis provides the most reliable results when performed correctly, because it provides statistically testable evidence for the convergence of the current literature. In addition, the sensitivity analysis, publication bias, and quality evaluation were carried out as a reference for the reliability and stability of the conclusions. Our cautious idea was that the brain abnormalities of DLB should be included, but not be limited to the results reported in this work. Systematic or even narrative reviews could represent important supplements. Third, a separate meta‐analysis of different symptom dimensions in DLB patients could not be conducted, since separate results of these potentially relevant variables were usually not reported. With sufficient data, a wider variety of subtype analysis based on the clinical characteristics of DLB beyond total DLB should be performed. Fourth, a subgroup meta‐analysis only including DLB patients that did not receive any type of DLB treatment could not be performed. Since previous reports showed that antidepressants,^123^ dopamine preparations,^124^ and cholinergic drugs^125^, ^126^ may alter imaging characteristics, further studies considering the medications of the DLB patients are necessary to confirm our results.

## 5 CONCLUSION

Overall, the present meta‐analysis suggests that the alterations of the brain structure and function in DLB might be specific and significantly different from AD. Co‐activated neural networks correspond to the FPN, VIS, and SN of HCs, suggesting that DLB might be abnormal in these networks. The integrity of the DMN in DLB patients provides a new observation to help in the clinical distinction between AD and DLB. The identified brain regions or networks might serve as a framework for future quantitative analysis of per‐subject image data. Such customized imaging indices might help the development of diagnosis, prognostic judgment, and targeted network regulation, thus improving the clinical management. However, a further study of the phenotype of DLB is necessary in order to comprehensively evaluate the neuroimaging features of DLB and its physiological significance. In addition, future early diagnosis and in‐depth understanding of DLB, AD, and other types of dementia will likely rely on multimodal approaches, through a combination of the mature imaging and some of the promising novel imaging modalities, such as molecular imaging and novel functional imaging.

## CONFLICT OF INTEREST

There are no conflicts of interest that need to be disclosed.

## Supporting information

Supplementary Material

## Data Availability

The data that support the findings of this study are available from the corresponding author upon reasonable request.
