# Supplementary material for: Neuroimaging alterations in dementia with Lewy bodies and neuroimaging differences between dementia with Lewy bodies and Alzheimer's disease: An activation likelihood estimation meta‐analysis
Source: CNS Neurosci Ther. 2021 Dec 6;28(2):183–205. doi: 10.1111/cns.13775 (PMC8739049; doi:10.1111/cns.13775)
Supplement: Supplementary file 1 — Supplementary Material [file CNS-28-183-s001.docx]

**Supplementary Material**

**Figure S1:** Step by step binary selection flowchart of literature search and selection strategy.


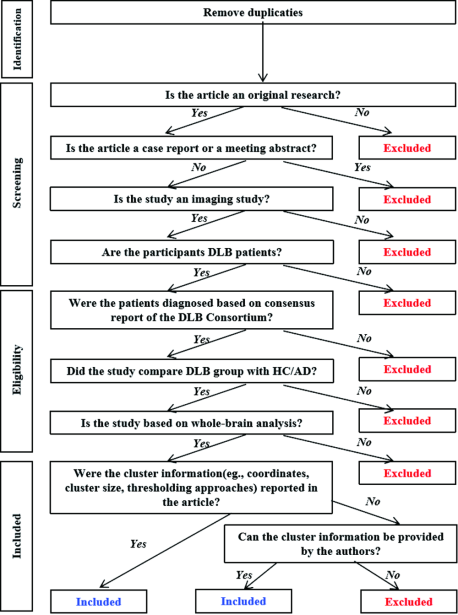


DLB = dementia with lewy body, HC = healthy control, AD = Alzheimer disease.

**Table S1:** Search terms for the systematic literature search

| **Database** | **Search terms** |
| --- | --- |
| PubMed | All Fields (“Magnetic Resonance Imaging”[Mesh] OR “Positron-Emission Tomography”[Mesh] OR “Tomography, Emission-Computed, Single-Photon”[Mesh] OR “MRI” OR “magnetic resonance imaging” OR “imaging” OR “neuroimaging” OR “brain imaging” OR “gray matter” OR “white matter” OR “voxel-based morphometry” OR “VBM” OR “voxelwise” OR “positron emission tomography” OR “PET” OR “single photon emission computed tomography” OR “SPECT”) AND (“ Lewy” OR “Lewy Body Disease”[Mesh]) |
| Web of Science | All Fields ( “MRI” OR “magnetic resonance imaging” OR “imaging” OR “neuroimaging” OR “brain imaging” OR “gray matter” OR “white matter” OR “voxel-based morphometry” OR “VBM” OR “voxelwise” OR “positron emission tomography” OR “PET” OR “single photon emission computed tomography” OR “SPECT”) AND (“Lewy” ) |
| OVID | Same as Web of Science |
| Science Direct | All Fields ( “MRI” OR “VBM” OR “fMRI” OR “PET” OR “SPECT” ) AND (“Lewy” ) |
| Cochrane Library | Same as PubMed |

| **Table S2: Quality assessment checklist (score 0/0.5/1 per item; total score out of 12)*** | |
| --- | --- |
| Category 1: Samples | |
|  | 1.Patients were evaluated prospectively, specific diagnostic criteria were applied, and demographic data were reported. |
|  | 2.Healthy controls were evaluated prospectively, psychiatric and medical illnesses were excluded. |
|  | 3.Important variables (e.g. age, sex, illness duration, onset time, medication status, comorbidity, severity of illness) were checked, either by stratification or statistically. |
|  | 4.Sample size per group > 10. |
| Category 2: Methods for image acquisition and analysis | |
|  | 5.Magnet strength at least 1.5 T (for MRI studies), or |
|  | Provide details of scanning thickness, voxel size and matrix size (for FET / SPECT studies). |
|  | 6.MRI slice thickness ≤ 3 mm (for MRI studies), or |
|  | Describe the use details of radioactive tracer (e.g. type, dose, distance scanning time) (for FET / SPECT studies). |
|  | 7.Whole brain analysis was automated with no a priori regional selection. |
|  | 8.Coordinates reported in a standard space. |
|  | 9.The imaging technique used was clearly described so that it could be reproduced. |
|  | 10.Measurements were clearly described so that they could be reproduced. |
| Category 3: Results and conclusions | |
|  | 11.Statistical parameters for significant, and important nonsignificant differences were provided. |
|  | 12.Conclusions were consistent with the results obtained and the limitations were discussed. |
| *When criteria were partially met, 0.5 points were awarded. | |

**Table S3:** Jackknife sensitivity analysis of the group of functional changes between DLB and HCs

| All studies but… | Right Inferior Parietal Lobule | Right Lingual Gyrus | Left Inferior Parietal Lobule |
| --- | --- | --- | --- |
| Ishii, K., 2007 | √ | √ | √ |
| Perneczky, R., 2007 | √ | √ | √ |
| Yong, S. W., 2007 | √ | √ | X |
| Teune, L. K., 2010 | √ | X | X |
| Ko, J. H., 2017 | √ | √ | √ |
| Liu, S., 2017 | √ | √ | X |
| Liguori, C., 2019 | √ | √ | X |
| Iizuka, T., 2020 | √ | √ | √ |
| Colloby, S. J., 2002 | √ | √ | √ |
| Firbank, M. J., 2003 | √ | √ | √ |
| Takahashi, R., 2010 | √ | X | √ |
| Takahashi, R., 2010 | √ | X | √ |
| Misch, M. R., 2014 | √ | X | X |
| Park, K. W., 2018 | √ | √ | √ |
| Total | 14 out of 14 | 10 out of 14 | 9 out of 14 |

**Table S4:** Jackknife sensitivity analysis of the group of structural changes between DLB and AD

| All studies but… | Left Parahippocampal Gyrus |
| --- | --- |
| Burton, E. J, 2002 | √ |
| Takahashi, R., 2010 | X |
| Watson, R., 2012 | X |
| Blanc, F., 2016 | √ |
| Heitz, C., 2016 | X |
| Roquet, D., 2017 | √ |
| Total | 3 out of 6 |
